# What to expect when you're evaluating healthcare improvement: a concordat approach to managing collaboration and uncomfortable realities

**DOI:** 10.1136/bmjqs-2014-003732

**Published:** 2015-04-02

**Authors:** Liz Brewster, Emma-Louise Aveling, Graham Martin, Carolyn Tarrant, Mary Dixon-Woods, The Safer Clinical Systems Phase 2 Core Group Collaboration & Writing Committee, Nick Barber

**Affiliations:** Department of Health Sciences, University of Leicester, Leicester, UK

**Keywords:** Quality improvement, Patient safety, Evaluation methodology, Quality improvement methodologies

## Abstract

Evaluation of improvement initiatives in healthcare is essential to establishing whether interventions are effective and to understanding how and why they work in order to enable replication. Although valuable, evaluation is often complicated by tensions and friction between evaluators, implementers and other stakeholders. Drawing on the literature, we suggest that these tensions can arise from a lack of shared understanding of the goals of the evaluation; confusion about roles, relationships and responsibilities; data burdens; issues of data flows and confidentiality; the discomforts of being studied and the impact of disappointing or otherwise unwelcome results. We present a possible approach to managing these tensions involving the co-production and use of a concordat. We describe how we developed a concordat in the context of an evaluation of a complex patient safety improvement programme known as Safer Clinical Systems Phase 2. The concordat development process involved partners (evaluators, designers, funders and others) working together at the outset of the project to agree a set of principles to guide the conduct of the evaluation. We suggest that while the concordat is a useful resource for resolving conflicts that arise during evaluation, the process of producing it is perhaps even more important, helping to make explicit unspoken assumptions, clarify roles and responsibilities, build trust and establish open dialogue and shared understanding. The concordat we developed established some core principles that may be of value for others involved in evaluation to consider. But rather than seeing our document as a ready-made solution, there is a need for recognition of the value of the process of co-producing a locally agreed concordat in enabling partners in the evaluation to work together effectively.

## Introduction

Meaningful evaluation has an essential role in the work of improving healthcare, especially in enabling learning to be shared.^1^ Evaluations typically seek to identify the aims of an intervention or programme, find measurable indicators of achievement, collect data on these indicators and assess what was achieved against the original aims.^2^ Evaluating *whether* a programme works is not necessarily the only purpose of evaluation, however: *how* and *why* may be equally important questions,^3^ ^4^ especially in enabling apparently successful interventions to be reproduced.^5^ Despite the potential benefits of such efforts, and the welcome given to evaluation by some who run programmes, the literature on programme evaluation has long acknowledged that evaluation can be a source of tension, friction and confusion of purpose:

[Evaluation] involves a balancing act between competing forces. Paramount among these is the inherent conflict between the requirements of systematic inquiry and data collection associated with evaluation research and the organizational imperatives of a social program devoted to delivering services and maintaining essential routine activities.^6^

Healthcare is no exception to the general problems characteristic of programme evaluation: the concerns and interests of the different parties involved in an improvement project and its associated evaluation may not always converge. These parties may include the designers and implementers of interventions (without whose improvement work there would be nothing to evaluate), the evaluators (who may be a heterogeneous mix of different professional groups - including health professionals and others - or academics from different disciplines) and sometimes funders (who may be funding either the intervention, the evaluation or both). Each may have different goals, perspectives, expectations, priorities and interests, professional languages and norms of practice, and they may have very distinct accountabilities and audiences for their work. As a result, evaluation work may—and in fact, often does—present challenges for all involved, ranging from practicalities such as arranging access to data, through conceptual disagreements about the programme and what it is trying to achieve, to concerns about the impartiality and competence of the evaluation team, widely divergent definitions of success and many others.^6^ Given that it is not unlikely these challenges will occur, the important question is how they can optimally be anticipated and managed.^7^ ^8^

This article seeks to make a practical contribution by presenting a possible approach to minimising the tensions. Specifically, we propose the co-production and use of a *concordat*—a mutually agreed compact between all parties, which articulates a set of principles to guide the conduct of the evaluation. The article proceeds in two parts. First, we identify the kinds of challenge often faced in the design, running and evaluation of an improvement programme in healthcare. Second, we present an example of the development of a concordat used in the evaluation of a major improvement project.

## Challenges in conducting programme evaluations

A now extensive literature has identified multiple challenges in programme evaluation, dating back to when the field began to develop formally during the 1960s.^9^ Challenges can arise at virtually every stage—from the design of the evaluation through its conduct and eventual publication—to the extent that ‘evaluation anxiety’ is a known phenomenon.^10^ Those being evaluated may be subjected to judgements about behaviour and outcomes against externally agreed targets. The detailed examination of individual, group and organisational practices may be experienced as risky and unpleasant, and strains in the relationships between the different parties may easily arise. These strains may, for example, relate to the goals of the evaluation; data management; the discomforts of being studied and disappointing or otherwise unwelcome results (box 1).

Box 1 **Areas of possible tension and challenge in programme evaluation identified in the literature**

- Securing full consensus on the specifics of evaluation objectives^34^
- Unpacking contrasting interpretations about what and who the evaluation is for^6^
- A desire on the part of evaluators to fix the goals for improvement programmes early in the evaluation process^35^
- Evolution of interventions (intentionally or unintentionally) during implementation^36^ and ongoing negotiation about evaluation scope in relation to implementation evolution^37^
- Fear of evaluation being used for performance management^15^
- Mismatched interpretations of stakeholders’ own role and other partners’ roles^12^ ^14^
- An interpretation of evaluators as friends or confidants, risking a subsequent sense of betrayal^16^
- A lack of shared language or understanding if some partners lack familiarity with the methodological paradigm or data collection tools being proposed^13^
- Conflicts between the burden of evaluation data collection and the work of the programme^2^
- Previous experiences of the dubious value of evaluation leading to disengagement with current evaluation work^17^
- Tensions between an imperative to feedback findings and to respect principles of anonymity and confidentiality^38^
- Encountering the ‘uncomfortable reality’ that a service or intervention is not performing as planned or envisaged and objectives have not been met^18^
- Negotiations with gatekeepers about access to complete and accurate data in a timely fashion
- A reluctance to share evaluation findings if they are seen as against the ‘organisational zeitgeist’^20^ or threaten identity and reputational claims^21^
- Pressure from partners, research sponsors or funders to alter the content or scope of the evaluation,^20^ or to delay their publication^22^

A critical first task for all parties is to therefore clarify what is to be achieved through evaluation. This allows an appropriate evaluation design to be formulated, but is also central to establishing a shared vision to underpin activity. This negotiation of purpose may be more or less formal,^11^ but should be undertaken. The task is to settle questions about purpose and scope, remembering that agreements about these may unravel over the course of the activity.^12^ Constant review and revisiting of the goals of the evaluation (as well as the goals of the improvement programme) may therefore be necessary to maintain dialogue and avoid unwarranted drift.

These early discussions are especially important in ensuring that all parties understand the methods and data collection procedures being used in the evaluation.^13^ A lack of shared language and understanding may lead to confusion over why particular methods are being used, generating uncertainties or suspicion and undermining willingness to cooperate. Regardless of what form it takes, the burden of data collection can be off-putting for those being evaluated and those performing the evaluation. If the evaluation itself is too demanding, there may be conflicts between its requirements and doing the work of the programme.^2^ For partner organisations, collecting data for evaluation may not seem as much of a priority as delivery, and the issue of who gets to control and benefit from the data they have worked so hard to collect may be difficult to resolve.

Even when agreement on goals and scope is reached early on and remains intact, complex evaluations create a multiplicity of possible lines of communication and accountability, as well as ambiguity about roles. Though the role of each party in a programme evaluation may seem self-evident (eg, one funds, one implements, one evaluates), in practice different parties may have mismatched interpretations both of their own role and of others’. Such blind spots can fatally derail collaborative efforts.^12^ The role of the evaluator may be an especially complex one, viewed in different ways by different parties.^14^ Outcomes-focused aspects of evaluation—aimed at assessing degree of success in achieving goals—may cast evaluators as ‘performance managers’.^15^ But the process-focused aspects of evaluation—particularly where they involve frequent contact between evaluators and evaluated, as is usually the case with ethnographic study—may make evaluators seem like friendly confidants, risking a subsequent sense of betrayal.^16^ Thus, evaluators may be seen as critical friends, co-investigators, facilitators or problem solvers by some, but also as unwelcome intruders who sit in judgement but do not get their hands dirty in the real work of delivering the programme and who have influence without responsibility.

Uncertainties about what information should be shared with whom, when and under what conditions may provide a further source of ethical dilemma, especially when unspoken assumptions and expectations are breached, damaging trust and undermining cooperative efforts. Evaluators must often abide by both the imperative to feedback findings to other stakeholders (especially, perhaps, the funders and clients of the evaluation) *and* to respect principles of anonymity and confidentiality in determining the limits of what can be fed back, to whom and in how much detail. For these reasons, role perceptions and understandings about information exchange (content and direction) need to be surfaced early in the programme—and revisited throughout—to avoid threats to an honest, critical and uncompromised evaluation process. This is especially important given the asymmetry that may arise between the various parties, which can lead to tensions about who is in charge and on what authority.

Sometimes, though perhaps not often, the challenges are such that implementers may feel that *obstructing* evaluation is more in line with their organisational interests. They may, for example, frustrate attempts to evaluate by providing inaccurate, incomplete or tardy data (quantitative or qualitative) or, where they are able to play the role of ‘gatekeeper’, simply deny access to data or key members of staff. A lack of engagement with the process may be fuelled by previous experiences of evaluation that was felt to be time-consuming or of dubious value.^17^

Tensions do not, of course, end when the programme and evaluation are complete, and may indeed intensify when the results are published. Those involved in designing, delivering and funding a programme may set out with great optimism; they may invest huge energy, efforts and resource in a programme; they may be convinced of its benefits and success and they may want to be recognised and congratulated on their hard work and achievement. When evaluation findings are positive, they are likely to be welcomed. Robust evidence of the effectiveness of an intervention can be extremely valuable in providing weight to arguments for its uptake and spread, and positive findings from independent evaluation of large-scale improvement programmes help legitimise claims to success. But not every project succeeds, and an evaluation may result in some participants being confronted with the uncomfortable reality that their service or their intervention has not performed as well as they had hoped.^18^ Such findings may provoke reactions of disappointment, anger and challenge: ‘for every evaluation finding there is equal and opposite criticism’.^19^

When a programme falls short of realising its goals, analysis of the reasons for failure can produce huge net benefits for the wider community, not least in ensuring that future endeavours do not repeat the same mistakes.^2^ But recognising this value can be difficult given the immediate disappointment that comes with failure. If the evaluation—and the resulting publications—does not present the organisation(s) involved in the intervention in a positive light, there may be a reluctance to ‘wash dirty linen in public’^2^ and resistance to the implications of findings,^20^ especially where they threaten reputation.^21^ Evaluators themselves may not be immune to pressures to compromise their impartiality. The literature contains cautionary examples of pressure from partners or research sponsors who wish to direct the content of the report or analysis,^20^ or coercion from funders to limit the scope of evaluation, distort results or critically delay their publication.^22^

## A possible solution: developing a concordat

By now it will be clear that challenges in conducting programme evaluation should be anticipated with a view to managing them (box 2). But how should this be done? Attempts to answer this question commonly include exhortations for stakeholders to commit to open dialogue and respect for other stakeholders, to have clear founding principles, a shared vision and transparent mechanisms for conflict resolution.^7^ ^23^ ^24^ While these are all important, guidance on how to achieve them in practice is limited. We propose that one promising solution lies in evaluation partners (evaluators, designers, implementers, funders and others) working together at the outset of a project to produce a concordat. It requires them to develop a set of principles to guide the conduct of the evaluation and agreeing to abide by these principles, consistent with the approach advocated by the Harvard Negotiation Project.^25^ We elaborate the rationale behind this proposal by drawing on our experience of developing a concordat for the evaluation of a large, multi-partner patient safety improvement programme.

Box 2 **Managing risks of conflict and tension in the evaluation of improvement programmes**

- All parties should agree on the purpose and scope of the evaluation upfront, but recognise that both may mutate over time and need to be revisited
- An explicit statement of roles may ensure that understandings of the division of labour within an evaluation—and the responsibilities and relationships that imply—are shared
- The expectations placed on each party in relation to data collection should be reasonable and feasible, and the methodological approach (in its basic principles) should be understood by all parties
- Clear terms of reference concerning disclosure, dissemination and the limits of confidentiality are necessary from the start
- All efforts should be made to avoid implementers experiencing discomfort about being studied: through ensuring all parties are fully briefed about the evaluation; sharing formative findings and ensuring appropriate levels of anonymity in reporting findings
- Commitment to learning for the greatest collective benefit is the overriding duty of all parties involved—it follows from this that all parties should make an explicit commitment to ensuring sincere, honest and impartial reporting of evaluation findings

The programme we discuss, known as Safer Clinical Systems Phase 2, was a complex intervention in which eight organisations were trained to apply a new approach (adapted from high-risk industries) to the detection and management of risk in clinical settings.^26^ The work was highly customised to the particularities of these settings. The programme involved a complicated nexus of actors, including the funder (the Health Foundation, a UK healthcare improvement charitable foundation); the technical support team (based at the University of Warwick Medical School), who designed the approach and provided training and support for the participating sites over a 2-year period; the eight healthcare organisations (‘implementers’) and the evaluation team (itself a three-university partnership led by the University of Leicester).

## Developing the concordat and its content

The evaluation team drew on the literature and previous experience to anticipate potential points of conflict or frustration and to identify principles and values that could govern the relationships and promote cooperation. These were drawn together into the first draft of a document that we called a ‘concordat’. The evaluation team came up with the initial draft, which was then subject to extensive comment, discussion, refinement and revision by the technical support team and funders. The document went through multiple drafts based on feedback, including several meetings where evaluators, technical team and funders came up with possible areas of conflict and possible scenarios illustrating tensions, and tested these against the concordat. Once the final draft was agreed, it was signed by all three parties and shared with the participating sites.

The first section of the concordat—‘goals and values’—sets out the core principles concerning the purpose of the activity (box 3). These were the constitutional foundations: they emphasised a shared, overarching goal—safer healthcare for patients—and committed all parties to adherence to this principle in all their interactions. In foregrounding these principles, the intention was to address the misconceptions that can occlude understanding of evaluation and to make explicit shared objectives.

Box 3 **The concordat in outline**

- *Goals and values*—outlining the partners and their commitment to the programme goal, shared learning, respect for dignity and integrity and open dialogue
- *Responsibilities of the evaluation team*—summarising the purpose of the evaluation, making a commitment to accuracy in representation and reporting and seeking to minimise the burden on partners
- *Responsibilities of the support team*—a synopsis of the remit of one partner's role in relation to the evaluation team and their agreed interaction
- *Responsibilities of participating sites—*outlining how the sites will facilitate access to data for the evaluation team
- *Data collection—*agreeing steps to minimise the burden of data collection on all partners and to share data as appropriate
- *Ethical issues*—summarising issues about confidentiality, data security and working within appropriate ethical and governance frameworks
- *Publications*—confirming a commitment to timely publication of findings, paying particular attention to the possibility of negative or critical findings
- *Feedback*—outlining how formative feedback should be provided, received and actioned by appropriate partners

The concordat then sets out the roles and responsibilities of each party, including, for example, an obligation to be even-handed for the evaluation team, and the commitment to sharing information openly on the part of the technical support team (box 3). The concordat also articulated the relationships between the different parties, emphasising the importance of critical distance and stressing that this was not a relationship of performance management. The concordat further sought to address potential disagreements relating to the measures used in the evaluation. Rather than delineate an exhaustive list of what those methods and data would be, the concordat sets out the process through which measures would be negotiated and determined, and made explicit the principles concerning requests for and provision of data that would underpin this process (eg, the evaluation team should minimise duplicative demands for data by the evaluation team, and the participating sites should provide timely and accurate data).

The values and ethical imperatives governing action and interactions were also made explicit; for example, arrangements around confidentiality, anonymity and dissemination were addressed, including expectations relating to authorship of published outputs. Principles relating to research governance and feedback sought both to mitigate unease at the prospect of evaluation while also enshrining certain inalienable principles that are required for high-quality evaluation: for example, it committed all parties to sharing outputs ahead of publication, but it also protected the impartiality of the evaluation team by making clear that they had the final say in the interpretation and presentation of evaluation findings (though this did not preclude other partners from publishing their own work). Importantly, the concordat sets out a framework that all parties committed to following if disputes did arise. These principles were invoked on a number of occasions during the Safer Clinical Systems evaluation, for example, when trying to reach agreement on measurement or to resolve ambiguities in the roles of the evaluation and support teams. The concordat was also invaluable in ensuring that boundaries and expectations did not have to be continually re-negotiated in response to organisational turbulence, given that the programme experienced frequent changes of personnel over its course.

## Challenges in developing and using the concordat

Of course, neither the process nor the outcome of the concordat for this evaluation was without wrinkles. Some issues arose that had not been anticipated, and some tensions encountered from the start of the programme continued to cause difficulties. These challenges were in some respects unique to this particular context, but may provide general lessons to inform future evaluation work. For instance, the technical support team was charged with undertaking ‘learning capture’, which was not always easy to distinguish from evaluation, and it proved difficult to maintain clear boundaries about this scope. Future projects would benefit from earlier clarification of scope and roles.

The concordat took considerable time to develop and agree—around 6 months—in part because the process for developing the concordat was being worked on at the same time as developing the concordat itself. One consequence of this was that the participating sites (the implementers) were only given the opportunity to comment rather than engage as full partners. Future iterations should attempt to involve all parties earlier. We share this concordat and its process of development in part to facilitate the speedier creation of future similar agreements.

## The concordat as a solution: how does developing a concordat support effective collaborative activity?

The development of a concordat makes concrete the principles underpinning evaluation as a collaborative activity, and the concordat itself has value as a symbolic, practical and actionable tool for setting expectations and supporting conflict resolution.

The concordat as a document provides mutually agreed foundational principles which can be revisited when difficulties arise. In this sense, the concordat has value as a guide and point of reference.^21^ It also serves a symbolic function, in that it signals recognition—by all parties—of the centrality and importance of collaboration and a shared commitment to the process of evaluation. Formalising a collaborative agreement between parties, in the form of a non-binding contract, has the potential to promote a cooperative orientation among the parties involved and build trust.^27^ ^28^ That the concordat is written and literally signed up to by all parties is important, as this institutionalisation of the concordat makes it less susceptible to distortion over time and better able to ensure that mutual understanding is more than superficial. Further, because it is explicitly not a contract, it offers a means of achieving agreement on core principles, goals and values separate from any legal commitments, and it leaves open the possibility of negotiation and renegotiation.

Much of the value in developing a concordat, however, lies in the *process* of co-production by all parties—a case of ‘all plans are useless, but planning is indispensable’. Though we did not directly evaluate its use, we feel that its development had a number of benefits for all stakeholders. First, rather than waiting for contradictions to materialise as disruptive conflicts that impede the evaluation, the process of discussing and (re)drafting a concordat offers an opportunity to anticipate, identify and make explicit differences in interpretations and perspectives on various aspects of the joint activity. Each party must engage in a process of surfacing and reflecting on their own assumptions, interpretations and interests, and sharing these with other parties. This allows difference and alternative interpretations to be openly acknowledged (rather than denied or ignored)—a respectful act of recognition and a prerequisite of open dialogue.^29^ ^30^ Thus, the production of the concordat acts as a mechanism for establishing the kind of open dialogue and shared understanding so commonly exhorted.

Second, by explicitly reflecting on and articulating the various roles and contributions of each party, the concordat-building process helps to foreground the contribution that each partner makes to the project and its evaluation, showing that all are interdependent and necessary.^7^ This emphasis on the distributed nature of contributions can help to offset the dominance of asymmetrical, hierarchical positionings (such as evaluator and evaluated, funder and funded, for example).^21^ It can therefore enable all those involved to see the opportunities as well as the challenges within an evaluation process, and reinforce a shared understanding of the value of a systematic, well-conducted evaluation.

## Conclusions

Programme evaluation is important to advancing the science of improvement. But it is unrealistic to suppose that there will be no conflict within an evaluation situation involving competing needs, priorities and interests: the management of these tensions is key to ensuring that a productive collaboration is maintained. Drawing on empirical and theoretical literature, and our own experience, we have outlined a practical approach—co-production and use of a concordat—designed to optimise and sustain the collaboration on which evaluation activity depends. A concordat is no substitute for sincere, faithful commitment to an ethic of learning on the part of all involved parties,^31^ and even with goodwill from all parties, it may not succeed in eliminating discord entirely. Nonetheless, in complex, challenging situations, having a clear set of values and principles that all parties have worked through is better than not having one.

A concordat offers a useful component in planning an evaluation that runs smoothly by providing a framework for both anticipating and resolving conflict in collaborative activity. This approach is premised on recognition that evaluation depends on collaboration between diverse parties, and is therefore, by its collective nature, prone to tension about multiple areas of practice.^32^ Key to the potential of a concordat is its value, first, as an institutionalised agreement to be used as a framework for conflict resolution during evaluation activity, and, second, as a mechanism through which potential conflicts can be anticipated, made explicit and acknowledged *before* they arise, thereby establishing dialogue and a shared understanding of the purpose, roles, methods and procedures entailed in the evaluation.

The concordat we developed for the Safer Clinical Systems evaluation (see online supplementary appendix 1) is not intended to be used directly as template for others, although, with appropriate acknowledgement, its principles could potentially be adapted and used. Understanding the principles behind the use of a concordat (how and why it works) is critical.^33^ In accordance with the rationale behind the concordat approach, we do not advocate that other collaborations simply adopt this example of a concordat ‘as is’. To do so would eliminate a crucial component of its value—the process of collective co-production. The process of articulating potential challenges in the planned collaboration, and testing drafts of the concordat against these, is particularly important in helping to uncover the implicit assumptions and expectations held by different parties, and to identify ambiguities about roles and relationships. All parties must be involved, in order to secure local ownership and capitalise on the opportunity to anticipate and surface tensions, establish dialogue and a shared vision and foreground the positive interdependence of all parties.

## Supplementary Material

Web appendix
