# Supplementary material for: What to expect when you're evaluating healthcare improvement: a concordat approach to managing collaboration and uncomfortable realities
Source: BMJ Qual Saf. 2015 Apr 2;24(5):318–24. doi: 10.1136/bmjqs-2014-003732 (PMC4413682; doi:10.1136/bmjqs-2014-003732)
Supplement: Web appendix [file bmjqs-2014-003732-s1.pdf]

Appendix 1: Concordat: Evaluation of Safer Clinical Systems

Version 2: 16 March 2012

Goals and values

1. The second phase of Safer Clinical Systems approach is a unique opportunity to learn about promising approaches to improving patient safety.
2. The partners in this programme are:
    a. The Support Team (Warwick Medical School);
    b. The participating sites:
    c. The Health Foundation;
    d. The Evaluation Team (University of Leicester, University of Birmingham, and the Armstrong Institute of Johns Hopkins University).
3. All partners involved in the Safer Clinical Systems programme share the same goal: that of making healthcare safer for patients.
4. All partners involved in the programme are committed to contributing to systematic learning, and to sharing that learning for the benefit of others. All partners are committed to be open about, and to learn from, challenges, difficulties and failures, as well as from successes.
5. All partners are committed to respecting the dignity and integrity of all stakeholders in the programme.
6. All partners are committed to open, respectful dialogue, and will avoid pursuing individual positions or interests. Any disagreement will be resolved through reference to explicit principles and not by imposition of individual will or personality.
7. The ultimate principal beneficiaries of the programme will be future patients and the health systems that serve them, not any individual partner in the programme.
8. All partners are committed to being guided by these principles, goals and values in the way they work with each other over the course of the programme.

Responsibilities of the Evaluation Team

9. The purpose of the evaluation is to:
    a. Provide a critical analysis of the Safer Clinical Systems approach, with the aim of generating generalisable lessons about the strengths and weaknesses of the approach, and suggesting how it may be optimised if it is deemed overall to be of value in improving the care of patients. The evaluation will provide independent evidence of the degree to which the approach improves reliability of systems in the eight participating sites, an understanding of the way in which it achieves this effect, and a comparative understanding of the impact of local context on this process.
    b. Provide an independent and impartial view of the effectiveness of the approach, using data from a number of sources, including data collected by the participating sites and the Support Team, as well as data collected directly by the Evaluation Team from the participating sites and other partners. This will include evaluation of the extent to which the approach has worked in individual participating sites, and evaluation of the role of the Support Team in providing programme-level input to support site-level progress.
    c. Generate a deep understanding of the experience of using the Safer Clinical Systems approach (including the role of tools and techniques) and their effectiveness, and make recommendations about how the approach might be subject to further testing at scale. This will build on and complement the work of

the Support Team to develop and validate the specific tools used in the programme.
   d. Provide some formative feedback during the course of the programme (see also paragraphs 40 and 42–46 below) to enable mid-course corrections and adaptations where appropriate, while avoiding becoming part of the intervention.

10. The evaluation starts from the position that Safer Clinical Systems is a highly promising and plausible approach. It is committed to making a thorough assessment of its likely value for patient safety, and cannot prejudge the outcomes of this assessment.
11. The Evaluation Team will provide a protocol for the evaluation to be agreed by the Health Foundation and the Support Team. Significant changes to the protocol, including methods and scope, will not be made without explicit agreement of these partners.
12. The Evaluation Team work under a solemn obligation to be even-handed, fair, truthful and accurate in their data collection, analysis, and reporting.
13. The Evaluation Team will always seek to minimise the burden created by their work for participating sites and the Support Team. They will take specific steps to ensure that they avoid imposing excessive burden (see paragraphs 16, 24 and 25 below).

Responsibilities of Support Team in relation to the evaluation process

14. Programme materials and data will be shared freely with the evaluators, unless there are good reasons not to share. Sharing of such materials for purposes of the evaluation does not transfer any intellectual property rights in the materials to the Evaluation Team.
15. The Support Team will keep the Evaluation Team informed of programme meetings, visits and events.
16. The Support Team will contribute to discussions about the choice of measures to be used in the programme, in order to ensure that the measures chosen are consistent with the programme theory and the aspirations for the programme, and do not impose too much of a burden on the participating sites.
17. The Support Team will share data on the measures collected by the sites with the Evaluation Team. The data will be provided to the Evaluation Team monthly.
18. The Support Team will provide data on progress to the participating sites across the whole programme with sites identifiable to themselves but other sites anonymised.
19. The Support Team will be willing to share their experiences of the challenges and successes in implementing the programme.
20. While formative feedback is not binding (see paragraph 45 below), the Support Team will be open to receiving formative feedback about programme and will consider, with the Health Foundation, whether midpoint corrections to the programme design are reasonable and feasible.

Responsibilities of participating sites

21. The participating sites will facilitate the Evaluation Team in undertaking interviews and ethnographic fieldwork.
22. The participating sites will contribute to discussions about choice of measures to be used in the programme, in order to ensure that the measures used are appropriate, reflect their priorities, are locally credible, and can be collected to a high standard of data accuracy. The participating sites will consider carefully and act on the advice of the Support Team in choosing measures.
23. The participating sites will submit data plans specifying the measures they will use and will respond constructively to comments on the plans provided by the Evaluation Team.
24. The participating sites will contribute data on the measures to the Support Team monthly.

Data collection

25. The Evaluation Team will take all possible steps to avoid collecting the same, or substantially similar, data twice: if data have already been provided to the Support Team, the Evaluation Team should not ask for them again.
26. The Evaluation Team will take all possible steps to reduce the burden on participating sites of any data collection they undertake for the purposes of the evaluation.
27. Data plans will be prepared by the participating sites. The Evaluation Team will provide a set of explicit principles that should be considered in selecting measures and writing data plans. These plans will be reviewed by the Evaluation Team against the principles. Feedback on the data plans will be agreed with the Health Foundation and the Support Team before being given to the teams.
28. Data on the measures should be submitted monthly to the Support Team by the participating sites, and then shared with the Evaluation Team.
29. Participating sites commit to providing data to the Support Team in a regular, timely fashion.
30. The Evaluation Team will share findings from fieldwork and interviews in the participating sites with the Support Team and the Health Foundation.
31. The Evaluation Team will respect the intellectual property rights of the Support Team in relation to the specific tools and the overall approach.

Ethical issues

32. The Evaluation Team will obtain the appropriate ethics and governance approvals for their work.
33. The Evaluation Team will take rigorous steps to ensure data security.
34. The Evaluation Team will develop and provide suitable information materials to explain about the evaluation for patients and NHS staff at the participating sites.
35. When conducting fieldwork in the participating sites, the Evaluation Team will fully respect the confidential nature of patients' personal data and will ensure that they do not inappropriately invade patients' privacy or cause other harms during ethnographic observations and other data collection.
36. The Evaluation Team will be sensitive to the ethical issues in conducting ethnographic and interview work in people's workplaces. The participating sites will be told that data collected by the Evaluation Team will be confidential *to the programme* (not just to the Evaluation Team), and as such may be shared with the other partners. On occasion it may be appropriate to identify particular individuals within the programme – for example if they may benefit from particular support, though as far as possible this will be avoided.
37. No data that could identify a particular individual will be disclosed outside the programme. All quotations and fieldwork notes will be anonymised before being published.

Publications

38. The Evaluation Team will write up and publish their findings in a timely way, and will ensure that all partners get the opportunity to see any manuscripts before publication so that they are informed before any findings appear publicly. In order to preserve the impartiality of the evaluation, the incorporation of changes suggested by other partners to manuscripts led by the evaluators will be at the discretion of the Evaluation Team.
39. Where appropriate, members of the participating sites, the Support Team, or the Health Foundation may be authors or members of writing committees on publications or presentations arising from the evaluation.

40. Where appropriate, the Support Team, the Health Foundation or members of the participating sites may publish or present their own findings without involvement of the Evaluation Team (with suitable acknowledgement if appropriate). The participating teams, the Support Team, and the Health Foundation may choose to establish a dissemination and publications committee which will agree its own terms outside of this concordat.
41. If the evaluation generates negative or critical findings, the Evaluation Team is under a duty to make them explicit. In this circumstance, the Evaluation Team will seek to maximise the benefits of the evaluation and reduce any risks to individuals or organisations in so far as this is consistent with maintaining the integrity, truthfulness and accuracy of the evaluation.
42. Any use of data by those outside the programme will be with the agreement of all partners that own the data.

Feedback

43. The Evaluation Team will provide regular feedback to the Support Team and the Health Foundation on emergent findings. This feedback will be provided with the aims of:
    a. making any necessary mid-course corrections or adaptations to the programme to ensure its success, including aspects of programme delivery that may need to be adjusted in light of early learning produced by the Evaluation Team;
    b. checking and refining emerging theory about how the programme works, and identifying the extent of consensus across programme members;
    c. demonstrating the accountability of the Evaluation Team and ensuring that the Evaluation Team are kept briefed on any changing priorities for the evaluation;
    d. providing evidence on progress in meeting the programme's objectives.

44. The Evaluation Team has no role in performance managing any aspect of the programme or its partners. No data produced by the evaluation can be used for punitive or disciplinary purposes by any partner.
45. There may be circumstances where the Evaluation Team identify major apparent problems. If this occurs, the Evaluation Team will have no role in managing examples of major problems.
46. The Support Team does not have a duty to act on formative feedback, but will remain committed to listening to feedback and giving it fair consideration.
47. During programme meetings and events, and in interim project reports, general feedback on the evaluation will be provided to the participating sites. The Evaluation Team will not generally provide feedback directly to individual participating sites, to ensure that the evaluation does not become part of the intervention, to ensure that the sites are not given conflicting or confusing information, and to maintain clear boundaries between the evaluation and the programme. Any individual feedback from the evaluation will generally be provided to the participating sites by the programme.
